# Transforming Opioid Poisoning Surveillance Through Novel Technologies: Rationale and Methodological Protocol for Applying Natural Language Processing to Emergency Department Data

**DOI:** 10.1111/dar.70117

**Published:** 2026-02-18

**Authors:** Ting Xia, Tina Lam, Joanna F. Dipnall, Jane Hayman, Richard Beare, Nadine E. Andrew, Amanda Roxburgh, Paul M. Dietze, Suzanne Nielsen

**Affiliations:** ^1^ Monash Addiction Research Centre, Eastern Clinical School Monash University Melbourne Australia; ^2^ School of Public Health and Preventive Medicine Monash University Melbourne Australia; ^3^ School of Medicine Deakin University Geelong Australia; ^4^ Victorian Injury Surveillance Unit, Monash University Accident Research Centre Melbourne Australia; ^5^ Peninsula Clinical School, School of Translational Medicine Monash University Melbourne Australia; ^6^ National Centre for Healthy Ageing Monash University Melbourne Australia; ^7^ Burnet Institute Melbourne Australia

**Keywords:** emergency departments data, natural language processing, opioid poisoning

## Abstract

**Introduction:**

Timely surveillance of opioid‐related harm is critical to inform public health responses and policy evaluation. In Australia, where prescription and illicit opioids remain a leading cause of unintentional drug‐induced deaths, emergency departments (ED) are a vital point of contact for acute opioid poisonings. Existing surveillance systems rely on structured coding, yet much relevant information is recorded in free‐text fields, leading to underreporting or misclassification. This limits opportunistic identification of emerging patterns and weakens the evidence base for evaluating policy reforms. We aim to improve surveillance accuracy by applying natural language processing (NLP) to routinely collected ED data.

**Methods:**

Using medical concept annotation tools, we will develop models trained on 15 years of Victorian Emergency Minimum Dataset (VEMD) records. These models will analyse both unstructured and structured fields to identify opioid poisoning presentations and be validated against a manually coded gold standard using standard performance metrics. In the second phase, we will incorporate additional unstructured clinical information such as discharge summaries from hospital electronic records, which are not available in the VEMD data, thereby allowing more comprehensive and accurate classification. Finally, we will assess the utility of NLP‐enhanced data in evaluating three major opioid policy changes.

**Discussion and Conclusions:**

This study is the first to apply NLP at large scale to Australian ED data for drug poisonings. By improving the accuracy and consistency of opioid poisoning identification, this approach can strengthen routine surveillance and better inform timely policy and health system responses without increasing the workload for clinical staff.

## 1 Introduction

Medication‐related harm is a leading cause of injury, and the third World Health Organization global patient safety challenge [1]. The World Health Organization reported an estimated 125,000 opioid‐related deaths globally in 2019 [2]. Non‐fatal overdoses are substantially more common, occurring nearly 30 times more frequently than fatal overdoses [3]. This has prompted a range of policies to restrict the supply of opioids and other high‐risk medications [4, 5]. Timely surveillance of opioid poisoning is critical to detecting shifts in trends early and evaluating the real‐world impact of opioid policies. Yet, current surveillance systems are limited in their ability to capture the complexity of opioid‐related harms, particularly those relying solely on structured administrative data.

Surveillance tools need to be timely, scalable and flexible enough to reflect changes in patterns of use, emerging substances and the unintended consequences of regulatory responses [6]. Emergency departments (ED) are key sentinel sites for monitoring opioid‐related harms, as they are often the first point of contact for individuals experiencing acute poisoning or overdose. In EDs, coding challenges related to reason for presentation are well‐recognised, as clinicians or triage nurses must select from a predefined shortlist from the International Classification of Diseases, Tenth Revision, Australian Modification (ICD‐10‐AM) [7]. As a result, codes are generally less accurate and often inadequate for identifying the true extent of opioid harm [8]. Previous studies based on ED data from Victoria, Australia, have demonstrated that nearly half of opioid‐related ED presentations are misclassified or entirely missed in structured diagnosis fields due to coding limitations or time constraints on clinical staff [9]. For example, ED opioid poisoning presentations have been commonly coded as ‘T50.9’ ‘Other and unspecified drugs, medicaments and biological substances’, or with the poisoning code ‘T40.6’ ‘Other and unspecified psychodysleptics’, both unrelated to opioid poisoning [10]. A previous study examining current clinician completed ICD‐10‐AM codes in Victorian EDs determined that for people who inject drugs, around half of ED opioid poisoning cases are not correctly coded [9]. Further, without manual recoding of ED data, key shifts such as from oxycodone to heroin are not detectable [11]. These coding inaccuracies can result in significant underestimation of opioid harms, prevent timely responses to emerging concerns, and weaken the quality of evidence used to inform clinical decision‐making, policy development and outcome evaluation.

Most clinically relevant information for classifying opioid‐related ED presentations is captured in unstructured free‐text fields. Currently, this rich source of information has not been analysed efficiently across large volumes of ED data for population‐level surveillance, posing a significant barrier to timely and accurate monitoring of opioid harms [12, 13]. Natural Language Processing (NLP), a subfield of Artificial Intelligence focusing on the interaction between computers and human language, offers a promising solution to the classification of opioid‐related ED presentations. NLP integrates computer science and linguistics to create technologies that can interpret and generate human language [14]. This field of Artificial Intelligence has progressed from predefined rules to extract or classify information to the more advanced machine learning models that learn to perform tasks from training data. By applying NLP techniques to the unstructured clinical free text within ED records, it may be possible to extract relevant concepts, identify patterns, and classify cases with greater precision. In recent years, several studies from the United States have demonstrated the value of NLP for detecting opioid involvement in clinical settings. For example, the U.S. National Centre for Health Statistics applied an enhanced opioid identification algorithm that integrates NLP to improve the detection of opioid‐related hospital encounters from the National Hospital Care Survey data, with approximately 20% of cases identified exclusively through NLP techniques [15]. Similarly, applying NLP to clinical notes has demonstrated robust performance in identifying patients with opioid use disorder or problematic opioid use [16, 17].

Despite these advances, NLP techniques have not been applied to opioid surveillance in Australia. Australia differs substantially from North America in its patterns of opioid use and harm. For example, illicit fentanyl use remains rare in Australia, but prescription opioid use is more prevalent [18]. The terminology used by clinicians, local slang and even the ICD‐10‐AM coding framework also present unique challenges. Therefore, NLP tools developed in other countries will not easily translate to Australian health system data without local adaptation and validation. The application of NLP to Australian ED data could transform the way substance‐related presentations are coded, monitored and understood. The potential for real‐time, low‐cost and scalable improvements in surveillance accuracy is possible, thereby enhancing the quality of data used for research, policy evaluation, and clinical planning and response. This potential could be realised without placing additional documentation burdens on frontline healthcare staff.

Between 2018 and 2020, a range of key policy changes were implemented in Australia to address the growing number of opioid‐related harms (e.g., codeine up‐scheduling and prescription monitoring) [4, 19]. Yet, inadequate coding of opioid harms in hospital systems makes it is challenging to accurately monitor opioid‐related harm, or understand whether policy interventions are achieving their intended effects. The overreaching aim for this project is to enable automated, on‐going, reliable surveillance of opioid‐related harms using ED data. Across three phases we will: (i) develop, deploy and validate a novel NLP model on 15 years of routinely reported emergency department injury data using the ‘description of event’ text field; (ii) determine if opioid coding can be enhanced using additional Electronic Health Records (EHR) data fields, such as discharge summary and pathology reports, beyond the standard reporting fields; and (iii) use the NLP enhanced data to examine the impact of recent opioid policies implemented in Australia on the rates of opioid poisoning related ED presentations.

## 2 Method and Analysis

### 2.1 Study Setting and Data Sources

This study is set in Australia, where the Emergency Care ICD‐10‐AM Principal Diagnosis Short List (EPD Short List) is used nationally to support consistent coding practices in EDs. This condensed list of codes and medical terms, derived from the broader ICD‐10‐AM classification, provides a nationally consistent approach to principal diagnosis reporting across Australian EDs and emergency services. This study will utilise two key data sources across two phases. Phase 1 will draw on 15 years (2009–2024) of Victorian Emergency Minimum Dataset (VEMD) injury data managed by the Victorian Injury Surveillance Unit (VISU). Victoria is one of only two Australian jurisdictions, alongside Queensland, that maintain a population‐level hospital injury surveillance system that includes free‐text descriptions. Phase 2 will use hospital ED data provided by Peninsula Health via the National Centre for Healthy Ageing Data Platform. Both these datasets are from Victoria, Australia's second most populous state, and the jurisdiction with the highest heroin harms [20]. Due to clinical and policy setting similarities and consistent standards in ED reporting across Australia, the findings of this study have the potential to inform emergency department surveillance practices nationally.

#### 2.1.1 VEMD—Routinely Collected Administrative ED Data

The VEMD injury data for this study will contain more than 4 million ED presentations, based on VISU's extraction of all injury‐related emergency department records submitted to the Victorian Department of Health for the 2009–2024 period. VISU maintains de‐identified records of all injury‐related presentations to public hospital EDs across Victoria. It includes data from 41 major public hospitals, representing the majority of emergency care delivered in the state and capturing nearly 97% of all injury‐related ED presentations statewide [21]. These data include detailed information on patient demographics, presentation characteristics, clinical diagnoses and outcomes. In addition, the VEMD contains a ‘description of event’ text field, which provides information about the injury event. Synthetic examples include entries such as ‘Patient OD on oxycodone’, ‘Brought to hospital by ambulance following fall; pain managed with valium and paracetamol’, ‘Hand laceration sutured; discharged with limited opioids for acute pain’ and ‘OD on sleeping pills …’. This is usually entered at triage with the aim of providing additional information about the event, to that contained in other data fields.

#### 2.1.2 Peninsula Health ED Data—Additional EHR Fields

**FIGURE 1:**
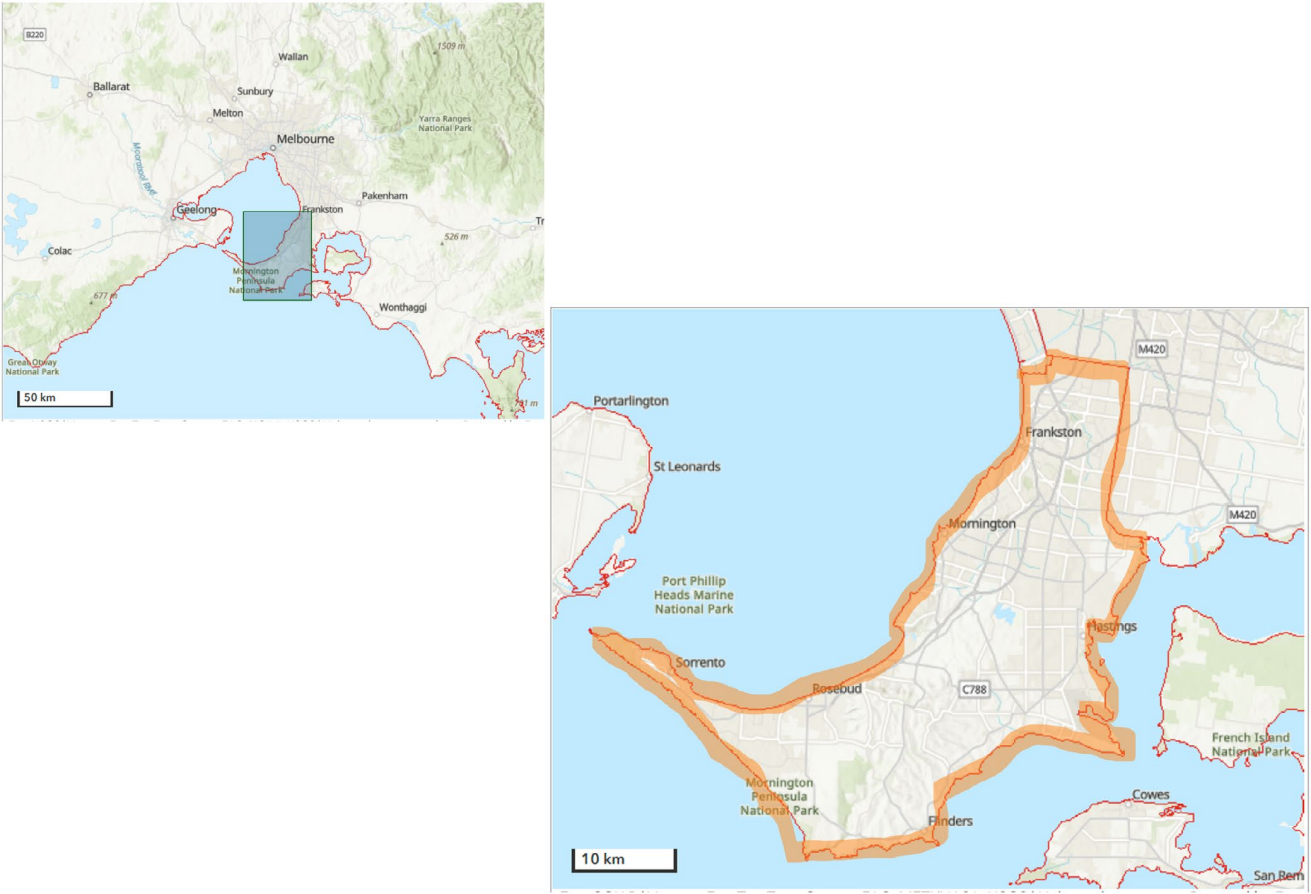
Frankston/Mornington Peninsula region. *Source*: https://maps.abs.gov.au/ .

The National Centre for Healthy Ageing (NCHA) Data Platform is a partnership between Monash University and Peninsula Health. Peninsula Health is the only public healthcare provider for the Frankston/Mornington Peninsula region serving a population of approximately 311,000 people through four hospitals and > 10 community and outpatient services [22] (Figure 1). The region contains both metropolitan and regional areas with broad socio‐economic diversity. The NCHA Data Platform brings together, internally links data, and curates data from all its administered services over a 10‐year period for over one million people to enable longitudinal tracking of healthcare utilisation, clinical outcomes and service delivery patterns [22]. Data domains include inpatient admissions, ED presentations from two acute care hospitals, outpatient visits, diagnostics, procedures, medication use and aged care assessments. The NCHA Data Platform has also brought together all clinical notes and documents into a common environment to support the application of NLP as described below [23].

Although Peninsula Health contributes data to the statewide VEMD, the VEMD includes only a limited free‐text ‘description of event’ field. The hospital‐level dataset contains richer clinical text fields (e.g., triage notes and discharge summaries) which can be used to extend and refine this model (Phase 2).

### 2.2 Natural Language Processing Tool

Named entity recognition and concept linking is the process of identifying meaningful terms in text and mapping them to standardised concepts, being a foundational step in clinical natural language processing. Modern approaches to named entity recognition, as implemented in Medical Concept Annotation Tool (MedCAT), employ deep learning techniques, particularly neural networks, to learn representations of text which reflect multiple meanings (are ‘high‐dimensional’), and context [24]. This allows MedCAT to capture semantic meaning beyond simple keyword matching, improving its ability to disambiguate terms based on context of use. MedCAT assigns concepts to words in text, based upon the context. Concepts are defined in the Unified Medical Language System (UMLS), which combines many ontologies (hierarchical representations of domain knowledge). Ontologies typically represent domain knowledge in hierarchical form. For example, in the SNOMED CT (an international standard for health terms) ontology (which is included in the UMLS) concepts for heroin, codeine, fentanyl and so forth are ‘children’ of the ‘parent’ concept “Morphinane alkaloid”, allowing automated combination of concepts into domain specific groups. Automated assignment of concepts to words in free text, defined in ontologies, allows structured data to be derived from unstructured text, in ways that are parallel to manual coding. For example, a successful annotation could assign different ontology concepts to the acronym “OD” depending on context, with possibilities including “overdose” (UMLS: C0579142), “Once Daily” (UMLS: C0556983) or “oculus dexter (right eye)” (UMLS: C0229089). Assigning ontology concepts to words performs data normalisation (by assigning synonyms to a single concept) and provides a structuring of domain knowledge.

This study will employ NLP techniques through CogStack, an open source platform developed by King's College London (*under an Apache 2 licence*) [24] that has been implemented within the electronic health record system at Peninsula Health. CogStack includes customisable facilities including the extraction of documents and searchable text from clinical systems, and tools to process large scale data. Within CogStack, we will use the MedCAT NLP framework, which combines a neural architecture with ontology‐based entity linking. MedCAT represents features using concept embeddings, which can be enhanced with contextual embeddings from transformer models including a variety of Bidirectional Encoder Representations from Transformers (BERT) models (e.g., ClinicalBERT, PharmBERT) [25]. The CogStack–MedCAT NLP framework has been thoroughly documented in previous publications [26]. These tools are prerequisites for applications of NLP methods to health service text data in Victoria. It has been used to facilitate several clinical research and service evaluation studies including enabling retrospective simulations of patient recruitment, supporting real‐time extraction of structured data from clinicians' free‐text notes, and performing real‐time text analysis to assist with clinical coding and the identification of clinical intent within unstructured documentation [27].

### 2.3 Phase 1. Algorithm Development and Validation on VEMD Injury Data

*Automated coding approaches using MedCaT are feasible and perform better than existing VEMD coding of opioid poisoning*.

The development of the NLP algorithm in Phase 1 will involve reviewing clinically relevant information from the unstructured text within the ‘description of event’ field in VEMD data. This process will be supported by experienced annotators. An initial set of annotation guidelines will be developed iteratively to create a structured set of instructions that help annotators consistently identify and label features in the free text field that indicate opioid poisoning, based on clinical definitions and coding standards. This process will be informed by a previously developed annotated pilot dataset of more than 5400 manually labelled opioid poisoning cases from 2009 to 2019 [13]. Cases will be identified if the name of an opioid drug or brand appears in the free‐text clinical notes and there is evidence of poisoning, indicated by either poisoning‐related cause of injury coding, relevant ICD‐10‐AM diagnosis codes (T400‐T509, T391 and T659), or text‐based terms such as “overdose”, “toxicity” or “poisoning.” Mentions of naloxone (e.g., Narcan) in the text are also considered indicative of opioid poisoning. Cases may be included even without a matching ICD code if manual review confirms opioid poisoning based on the text. Team members will document ambiguous cases in a shared spreadsheet, which will be reviewed collectively during calibration meetings. Inter‐annotator agreement will be regularly assessed using Cohen's kappa. Disagreements will be resolved by discussion and consensus, with final decisions made by a team of senior researchers and clinicians. The data will be randomly split into 75:25 training: test data, stratified by positive [opioid poisoning cases] to negative [non‐opioid poisoning cases] with an appropriate data‐level (e.g., oversampling in the minority class) or algorithm‐level (e.g., class weights) technique used during the training process to mitigate class imbalance.

The NLP development will proceed in two stages. First, we will automate the existing rule‐based approach, which uses predefined sets of keywords and logic rules (see Tables S1 and S2, Supporting Information) to mimic the manual classification process previously applied to the full 15 years of VEMD data, including multiple labels per case, such as general opioid poisoning and opioid type specified poisoning (e.g., oxycodone‐related, heroin‐related or multiple opioid exposures). This formalisation of the rule‐based classification process will allow scaling of the decision logic underlying human classifications. In the second stage, CogStack's MedCAT, will be trained to detect relevant clinical concepts and relationships using both ICD‐10‐AM poisoning codes and custom concepts designed to capture context‐specific opioid involvement. Multi‐label classification models, including logistic classifier, Support Vector Machine or Random Forest, will be trained using Cogstack annotations to automate the identification of opioid poisonings. These models will generate the same relevant labels per case as the rule‐based approach, enabling direct comparison. Discriminative ability will be assessed using the confusion matrix and a variety of key performance metrics (e.g., balanced accuracy, per class metrics (precision, recall, specificity Brier Score), macro averaged F1‐Score, Mathews Correlation Coefficient, area under the precision‐recall curve). Where applicable, 95% confidence intervals will be calculated via non‐parametric bootstrapping (e.g., 1000 resamples) to quantify uncertainty.

To compare the rule‐based model and the NLP‐based models, we will apply both approaches to the same test set and evaluate their classification outputs using the same performance metrics.

Once validated, the final NLP algorithm will be applied to the complete VEMD data (all injuries) from 2009 to 2024. To ensure quality assurance, a random sample of 1000 NLP‐identified cases will be manually reviewed to confirm classification accuracy. The output will then be used to generate a comprehensive dataset of ED presentations due to opioid poisoning over the 15‐year period.

### 2.4 Phase 2. Extend Coding Accuracy Using Advanced NLP Approaches with Hospital‐Held Data

Hypothesis 2 *That the inclusion of additional clinical documents will improve coding accuracy*.

In the second phase, this project expands upon the phase 1 NLP approach by incorporating additional unstructured clinical text associated with ED presentations from the Peninsula Health EHR system held within the NCHA Data Platform from 2009 to 2024. This phase aims to refine and extend opioid poisoning classification by leveraging additional clinical detail not available in the VEMD data. To support the development of a robust NLP model, we will first undertake a clinician‐driven process to identify and prioritise structured and unstructured EHR fields that can reliably indicate opioid poisoning episodes. These may include triage notes, clinical notes, discharge summaries, pathology reports, medication administration records and other relevant documentation. Fields will be iteratively reviewed and selected based on their clinical utility, consistency across settings, and availability within the data environment. Using the selected fields, we will first undertake a gold‐standard annotated dataset by manually classifying 500 ED presentations as opioid‐related or not, and where possible, by opioid type. Annotation will be conducted by trained clinical nurses using a clinical labelling’ tool developed for previous projects. This tool enables manual coding of clinical concepts across collections of ED‐related documents and allows coders to link structured fields to specific text spans. It also supports auditing and refinement of NLP outputs by presenting prefilled forms for validation.

A multi‐label classifier will then be trained using the annotated dataset, applying standard data‐splitting and cross‐validation techniques to evaluate performance as in Phase 1. Another key objective of this phase is to assess the incremental value of different unstructured documents to the performance of the NLP classifier. We will begin by training models using individual clinical documents (e.g., discharge summary), then progressively incorporate additional clinical documents including triage notes, clinical notes, pathology results and treatment pathway records, to quantify performance improvements at each step. Various regression‐based classification models, including logistic regression, multinomial logistic regression, Support Vector Machine or Random Forest, will be explored and compared to identify the most accurate and generalisable approach for multi‐label prediction. Performance metrics, including sensitivity, specificity, PPV, NPV, F1 score and AUC, will be reported. The best‐performing model will then be employed to identify opioid poisonings within all ED presentations at Peninsula Health. To assess generalisability, the final model at each step will be externally validated at a second hospital site, Alfred Health, where CogStack is also implemented. As a leading metropolitan health service in Melbourne, Alfred Health serves a larger and more complex patient population than Peninsula Health, with a broader metropolitan catchment area and a more diverse dataset. Subject to the availability of comparable unstructured data fields, this external validation will confirm its applicability beyond the original training setting.

### 2.5 Phase 3 Determine Policy Impacts on Opioid and Other Harm

Hypothesis 3 *Improved coding will enable the identification and monitoring of intended and unintended opioid harms that may have changed following the implementation of pharmaceutical opioid policies*.

The final phase of this project will determine whether enhanced coding can lead to clearer evidence of policy outcomes when compared to the routine method where opioid poisonings are identified only using ICD‐10 codes. This phase will focus on three opioid policies recently implemented in Australia: codeine rescheduling to a prescription only medicine (2018) [28]; mandated prescription drug monitoring programme implementation (April 2020) [4]; and reduced opioid pack sizes (July 2020) [5]. The study population will comprise patients who present to ED with an opioid‐related harm, as identified using standard ICD‐10‐AM coding, and compared across three datasets: (i) structured VEMD injury ED data without NLP coding; (ii) NLP‐coded VEMD injury ED data; and (iii) enhanced NLP‐coded hospital ED data (see Table 1 for a summary of study datasets used for each phase).

**TABLE 1:** Summary of datasets.

	1. Structured VEMD injury ED data without NLP coding	2. NLP‐coded VEMD injury ED data	3. Enhanced NLP‐coded Peninsula Health and Alfred Health ED data
Description	Standard Victorian ED data, deidentified and routinely suppled to government for monitoring	Victorian ED visits to hospitals following NLP coding	Complete ED patient records from Peninsula (catchment > 300,000) and Alfred Health (catchment > 770,000)
Role of dataset	Development and validation of NLP coding (Phase 1)	Policy analysis (Phase 3)	Determine if coding accuracy can be improved using additional clinical documents (Phase 2 & 3)
Variables	Demographics, month of presentation, day of the week presented, arrival mode, triage category, separation mode, ICD‐10‐AM diagnosis, ‘description of event’ free‐text field, cause of injury, human intent	Same as standard ED data with additional opioid variable (identifying opioid type) and ICD‐10‐AM coding as determined by NLP	Standard ED data in addition to clinical notes, discharge records and other clinical documents to determine enhanced ICD 10‐AM NLP coding

Abbreviations: ED, emergency department; NLP, natural language processing; VEMD, Victorian Emergency Minimum Dataset.

### 2.6 Analysis

We will first conduct the policy impact analyses using the full VEMD data, comparing outcomes based on the structured data and NLP‐coded data. The full 15 years of VEMD data will be used to examine long‐term trends in opioid poisoning. For each policy, we will specifically compare incidence rates in the 2 years before and after implementation to evaluate policy impacts. This will allow us to assess the overall impact of enhanced case identification on policy evaluation at the population level. Following this, additional analyses will be conducted using NLP‐coded and uncoded subsets of VEMD data specific to Peninsula Health and Alfred Health from Phase 1, enhanced NLP‐coded Peninsula Health and Alfred Health ED data from Phase 2. This step will enable us to determine whether the impacts observed in the statewide analysis are consistent across different local contexts and health service settings, and whether NLP offers similar improvements in case identification at the individual hospital level.

Advanced analytical methods will be employed, including Interrupted Time Series and Difference‐in‐Differences analyses. These are considered among the most powerful quasi‐experimental designs for evaluating natural experiments like policy change where data have been collected at regular intervals before and after an event [29]. The primary outcomes of interest for this analysis are changes in the rates of ED presentations related to prescription opioid poisoning. Monthly rates will be estimated using the population of Victoria as the denominator. The observation period will include 24 months before and 24 months after each policy change, with data aggregated by calendar month. As the PDMP and pack‐size reduction policies were implemented over a two‐month period, we will define the policy intervention window from 1 April 2020 to 1 June 2020. If statistically significant, harmonic terms will be incorporated into the models to account for seasonality. Sensitivity analyses with varying lag periods will be performed to address autocorrelation and assess the robustness of the primary findings. Additional regression analyses will be conducted to examine outcomes across specific subpopulations, including age and sex. To assess the improvement in case identification, we will compare the accuracy of each dataset by evaluating the proportion of correctly classified opioid‐related presentations, using a manually reviewed random sample as the reference standard. Nonparametric statistical tests for classifier comparison will be used to formally assess differences in coding performance across the datasets. This phase will provide critical insights into the role of NLP‐enhanced data in strengthening the evaluation of opioid policy impacts. By replicating each policy evaluation across the two NLP‐enhanced datasets developed through Phase 1 and Phase 2, we will quantify the incremental value added by each coding approach.

**FIGURE 2:**
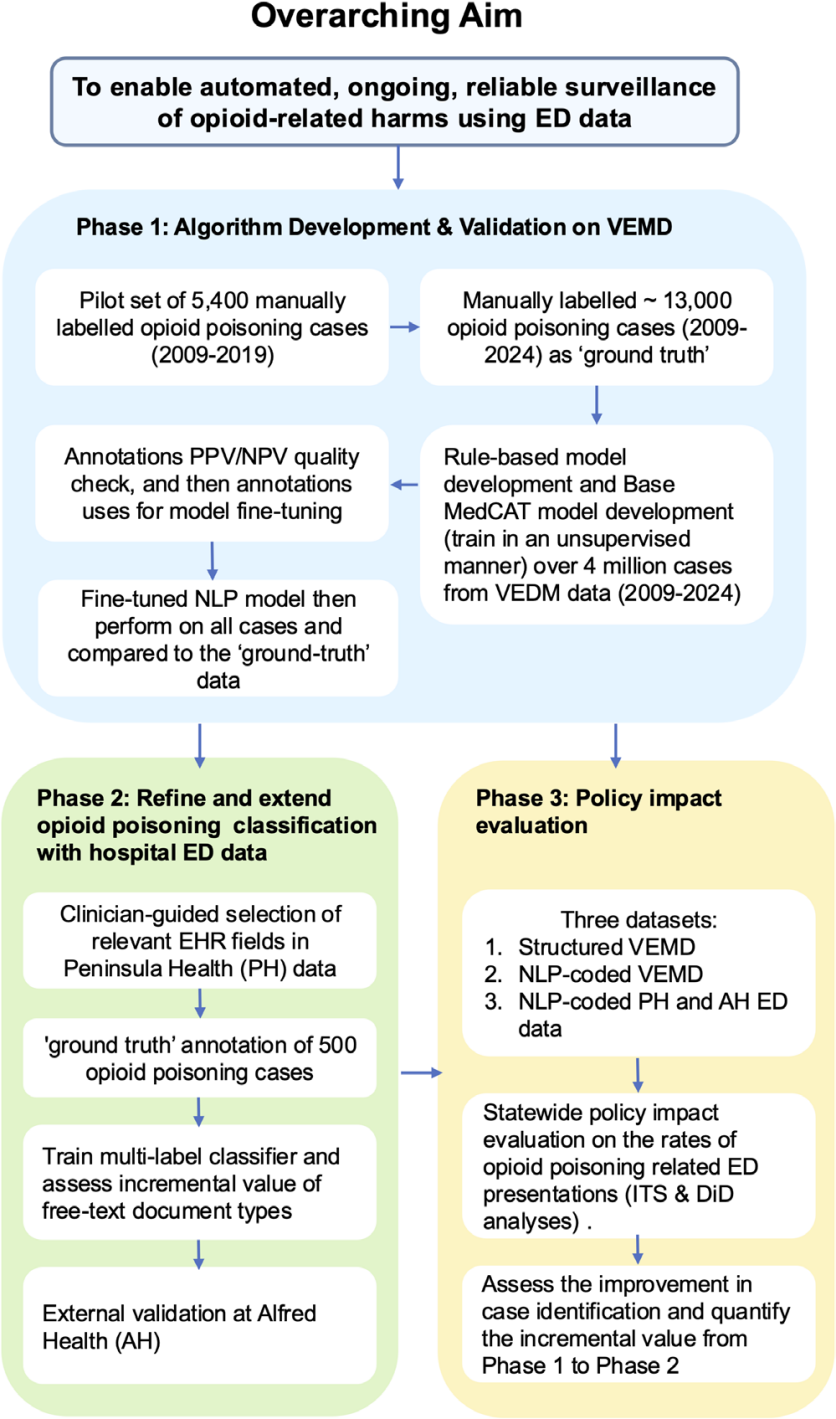
Data flow and study design. Abbreviations: ED, emergency department; EHR, electronic health record; NLP, natural language processing; VEMD, Victorian Emergency Minimum Dataset.

The overall data flow and study design is visualised in Figure 2.

## 3 Discussion

### 3.1 Strengths

This study presents several methodological and practical strengths that will innovatively enhance the use of health administrative data for research and inform policy development. First, this will be the first application of applying NLP coding approaches to enable low cost, yet accurate routine coding of ED data, addressing long‐standing challenges in the underreporting and misclassification of opioid‐related harms in routinely collected health data in Australia. Second, the NLP platform used in this study, CogStack, has a proven national and international track record of success [23, 27, 30, 31] and provides a robust technological foundation for this work. The integration of the NLP platform into Australian hospital systems, such as Peninsula and Alfred Health, provides a timely and proven technological foundation for monitoring, research and evaluation. CogStack enables sensitive unstructured EHR data to be analysed within the secure environment of the hospital or university, rather than transferring it externally [24]. This approach will minimise data movement and strengthen privacy protections, helping overcome common ethical and information governance barriers to using unstructured data for research. Another key strength of this project is that the NLP algorithm will be developed and validated using multiple complementary datasets, including state‐wide, population‐level ED data, as well as detailed hospital‐level datasets that integrate multiple sources of free‐text clinical information. This approach ensures that the algorithm is both robust and generalisable, and capable of performing accurately across diverse healthcare settings and data environments. However, we acknowledge that implementing these methods in new hospital systems requires a learning curve and dedicated resources, including staff training and IT infrastructure. Lastly, this project will enable detailed studies of policy interventions that aim to reduce harm with specific opioids (e.g., to assess the effects of reduced pack sizes on poisonings seen in EDs). This project will provide the first comprehensive evidence on the impact of these key opioid policy changes and will inform targeted mitigation strategies that are needed to address unintended harms. Understanding the impact of regulatory changes is increasingly urgent given rising harms, including suicide and self‐harm, associated with non‐opioid medicines such as gabapentinoids, antipsychotics and benzodiazepines. Our approach has the potential to be applied nationally as a framework to monitor harm and evaluate future policy changes or harm reduction interventions. Insights from this project also have the potential to advance international monitoring methods, addressing gaps in opioid and other substance surveillance, as well as a range of injuries currently undercaptured by existing coding systems.

### 3.2 Limitation

While NLP presents a promising approach for enhancing the identification and coding of opioid‐related emergency department presentations and hospital admissions from unstructured clinical data, there are several potential limitations that may impact its accuracy and generalisability. First, the challenges of identifying opioid‐related presentations often require understanding ambiguous clinical context. For example, the presence of terms like “overdose” can imply opioid poisoning but these terms can be used for any drug or refer to a previous event. Such terms may be misinterpreted and lead to false positives or false negatives. Free‐text observations may also mention opioids in a non‐causal way (e.g., history of use, discharge of use), and therefore it may be difficult to be certain that the opioid was causally associated with the presenting event. We will use iterative annotation, model training informed by gold‐standard data, and manual validation of random samples to maintain accuracy to mitigate this risk. Second, clinical free text information documentation can be significantly varied in detail, vocabulary, and structure across datasets, clinicians and hospitals. This heterogeneity could potentially affect the NLP model in its ability to capture relevant concepts consistently. To mitigate the impact of this heterogeneity, we will apply text normalisation, mapped synonyms to standardised medical vocabularies, and fine‐tune a transformer‐based NLP model on our local dataset. Third, incomplete reporting or missing data can limit the scope and effectiveness of the NLP system. Fourth, since CogStack is typically deployed within individual organisational units, data cannot be easily shared across institutions, limiting opportunities for cross‐site model training, validation, and benchmarking unless additional governance arrangements are established. Last, while the NLP techniques enable potentially real‐time and scalable surveillance without adding documentation burden to frontline clinicians, on‐going manual validation remains essential to maintain accuracy. Incorporating periodic calibration and feedback from clinicians can further enhance the reliability and responsiveness of NLP‐based surveillance systems as they are closest to patient care and have extensive experience in documentation practices.

### 3.3 Ethics and Dissemination

The study has received approval from the Monash University Human Research Ethics Committee (ID 76744). Study findings will be disseminated through peer‐reviewed journal publications.

## 4 Conclusion

This study aims to improve the identification of opioid‐related harms in Australia by enhancing the accuracy of coding in electronic health records using NLP approaches. By reducing misclassification and under coding of opioid presentations, our methods will generate more reliable evidence on the impacts of key opioid supply‐side policies and enable detection of emerging patterns of harm, including unintended consequences such as shifts to illicit opioids such as heroin. While developed for opioids, these methods have the potential to be adaptable to other high‐burden substances (e.g., alcohol, stimulants) and injury‐related harms, supporting broader surveillance efforts. The project also provides a foundation for future integration into national monitoring systems and linkage with mortality or toxicology data, enabling improved monitoring and informing evidence‐based policy and public health interventions.

## Author Contributions

Ting Xia and Suzanne Nielsen conceptualised and received funding for the study with chief and associate investigators (Joanna F. Dipnall, Jane Hayman, Tina Lam, Nadine E. Andrew, Richard Beare, Paul M. Dietze and Amanda Roxburgh). The original draft was produced by Ting Xia, Joanna F. Dipnall, Jane Hayman, and Tina Lam. All authors were involved in manuscript revision and jointly took responsibility for all aspects of the work.

## Funding

This work was supported by the Australian National Health and Medical Research Council (NHMRC, GNT2037997). SN is the recipient of an NHMRC Investigator Grant (#2025894).

## Conflicts of Interest

The authors declare no conflicts of interest.

## Supporting information


**Data S1:** Supporting Information.

## Data Availability

Data sharing not applicable to this article as no datasets were generated or analysed during the current study.
