# Supplementary material for: Transforming Opioid Poisoning Surveillance Through Novel Technologies: Rationale and Methodological Protocol for Applying Natural Language Processing to Emergency Department Data
Source: Drug Alcohol Rev. 2026 Feb 18;45(2):e70117. doi: 10.1111/dar.70117 (PMC12917345; doi:10.1111/dar.70117)
Supplement: Supplementary file 1 — Data S1: Supporting Information. [file DAR-45-0-s001.docx]

| Term category | Terms |
| --- | --- |
| Opioid term | Opioate, opiots, opiate, opiod, opioid, opipoid, opiate, opiaite, opialte, opium, pain killers, pain meds, heroin, herion, heroi, herioin, heriod, herorin, smack, heronin, herron, heroin, herronin, heroin, heroin, heoin, hereoin, heoird, herino, heron, heropin, herouin, herpin, herrion, heroin, heroi, methadone, methodone, methad, methodine, biodone, methadone, mentadone, menthadone, oxycodeine, oxycodeine, oxycontin, oxycontin, oxycintin, oxyc, endone, oxynorm, targin, oycontin, codeine, codene, codei, codeine, codie, codine, mersyndol, mersynd, mersyndal, aspalgin, nurofen plus, demazin, codeine, panafen, panafeine, hydromorphone, hydromorphine, hydromorhine, hydromorph, dilaudid, junista, jurnista, Vicodin, Lortab, lorcet, hycodan, vicoprofen, fentanyl, fentynal, durogesic, buprenorphine, bupra morhine /morphine), bupre (morphine/nophrine/norp/norphone/norphrine), buprenorphine, buprimorphine, bupromorphine, buprenorphine, norspan, suboxone, subox (suboxine soboxon), subutext, temgesic  Sublocade, buvidal, pethidine, pethidine, meperidine, Demerol, demarol, palexia, palexa, palexi, dextropoxphene, dextropropoxyphene, dextroproxyphene, digesic, capadex, paradex, doloxene, darvon, propoxyphene, pandene, panadol forte, panadiane, pandaniene, panadeien, panadei, codalgen forte, Codalgin, comfarol/comfral, panafen, pan forte, codapane, , ms mono, kapanol, kapanol, ordine, morph, tramadol, tramedo, lodam, tramal, zydol, maxitram, marol, zamadol, tramulief, tramquel |
| Naloxone term | naloxone, Narcan, narcain, naracn |
| Poisoning term | Overdose, over dose, od, o/d, o/dose, poisoning, toxicity |
| Poisoning ICD-10 codes | ‘T40’ to ‘T509’ (all drug poisoning codes)  ‘T659’ (Toxic effect of unspecified substance) |
| Poisoning category (VEMD internal coding) | Poisoning-medication (code 17)  Poisoning- other or unspecified (code 18) |

Supplementary

Table S1. Dictionaries of opioid use disorder and negation terms, and additional specialized terms, which were combined via parsing rules to form search phrases.

Table S2. Parsing rules defining the combinations of dictionary terms used in the identification of opioid poisoning

| Parsing rules | Rule contracture |
| --- | --- |
| Rule 1 | opioid term + poisoning term |
| Rule 2 | opioid term + poisoning ICD-10 codes |
| Rule 3 | opioid term + Poisoning category (VEMD internal coding) |
| Rule 4 | opioid term+ naloxone term |
